# Chromium Speciation by HPLC-DAD/ICP-MS: Simultaneous Hyphenation of Analytical Techniques for Studies of Biomolecules

**DOI:** 10.3390/ijerph20064912

**Published:** 2023-03-10

**Authors:** Vitória Aparecida Procópio, Rodrigo Mendes Pereira, Camila Neves Lange, Bruna Moreira Freire, Bruno Lemos Batista

**Affiliations:** Center for Natural and Human Sciences, Federal University of ABC, Santo André 09210-580, SP, Brazilbruna.freire@ufabc.edu.br (B.M.F.)

**Keywords:** Cr(VI), Cr(III), [Cr(III)-EDTA]^−^, HPLC-DAD/ICP-MS, biomolecules

## Abstract

The first element legislated adopting chemical speciation was chromium (Cr) for differentiation between the highly toxic Cr(VI) from the micronutrient Cr(III). Therefore, this work aimed to develop a new analytical method through the coupling of High-Performance Liquid Chromatography with Diode-Array Detection (HPLC-DAD) with inductively coupled plasma mass spectrometry (ICP-MS) to obtain molecular and elemental information simultaneously from a single sample injection. In the first step, a low-cost flow split made of acrylic was developed aiming at optimally directing the sample to the detectors, enabling the HPLC-DAD/ICP-MS coupling. After the extraction of Certified Reference Materials (CRM of natural water NIST1640a and sugar cane leaf agro FC_012017), the recoveries determined by ICP-MS were 99.7% and 85.4%, respectively. Then, the method of HPLC-DAD/ICP-MS was applied for real samples of the CRMs. The presence of possible biomolecules associated with Cr(III) and Cr(VI) species was evaluated, with the simultaneous response detection of molecular (DAD) and elementary (ICP-MS) detectors. Potential biomolecules were observed during the monitoring of Cr(VI) and Cr(III) in sugar cane leaves, water samples and a supplement of Cr picolinate. Finally, the article also discusses the potential of the technique applied to biomolecules containing other associated elements and the need of more bioanalytical methods to understand the presence of trace elements in biomolecules.

## 1. Introduction

Chromium (Cr) can occur naturally in the environment in the minerals chromite, hematite and magnetite. In addition, the main route of anthropogenic exposure to Cr occurs by contaminants generated by industrial processes such as metallurgy, electroplating, foundry, in the manufacture of steel, cement, batteries and chemicals in general used in paints, dyes, pigments and tanneries, in addition to the burning of fossil fuels, waste incineration, dumps, sanitary landfills and phosphate fertilizers [1,2,3].

More than 90% of Cr production worldwide is concentrated in seven countries, including India, Brazil and South Africa [3]. The World Health Organization (WHO) recommended that drinking water should contain a maximum concentration of 0.05 mg L^−1^ of total Cr, the same limits established by the Council of the European Union and Brazilian regulations. However, none of these regulations considers the chemical species of Cr (Cr(VI) and Cr(III)) [2,4].

Chromium (VI) is a highly toxic species, considered mutagenic and classified as a Group 1 substance (carcinogenic to humans) by the International Agency for Research on Cancer (IARC) [5]. On the other hand, Cr(III) is considered a micronutrient, but there are still doubts about its essentiality. According to the literature, Cr(III) in trace concentrations is important for the metabolism of carbohydrates, proteins and lipids [2,6]. According to IARC, Cr(III) belongs to Group 3 (not classifiable with respect to carcinogenicity in humans) [7]. Therefore, methods of analysis for Cr, regarding not only Cr(III)/Cr(VI) but also biomolecules containing Cr, are still necessary to improve the knowledge about this element and its role in living organisms.

The simultaneous coupling of High-Performance Liquid Chromatography (HPLC) with molecular detectors (such as the Electro-Spray Ionization Mass Spectrometer (ESI-MS) or Ultra-Violet/Visible Diode Array Detector (UV-Vis DAD)) and elemental detectors (such as the Inductively Coupled Plasma Mass Spectrometer (ICP-MS)) can be used for the identification and quantification of hundreds of natural biomolecules containing chemical elements in their composition [8,9,10,11]. This high-level hyphenated technique enables the determination of elements in transport mechanisms in plants, since the elemental species linked to organic molecules are relatively stable, allowing then to be properly separated by liquid chromatography [12]. Therefore, the present work aimed to develop a simultaneous hyphenation by coupling HPLC with DAD and ICP-MS detectors to obtain molecular and elemental information, respectively, from a single injection of sample containing different chemical forms of Cr.

## 2. Materials and Methods

### 2.1. Reagents and Instruments

For all tests, ultrapure water was used (resistivity 18.2 M Ω.cm Master System All, Gehaka, São Paulo, Brazil). The standard calibration solutions of Cr species were prepared: (i) Cr(VI) from a 100 mg L^−1^ Cr(VI) standard solution (AccuSPEC—SCP Science, Baie-d’Urfé, QC, Canada) and (ii) Cr(III) as chloride salt hexahydrate (CrCl_3_ 6H_2_O—Sigma Aldrich 98% purity, Burlington, MA, USA) were dissolved in an aqueous solution containing Ethylenediamine tetra-acetic acid (EDTA, Sigma Aldrich 99.995% purity, USA), forming [Cr(III)-EDTA]^−^ complex. An ammonium nitrate salt (NH_4_NO_3_, Sigma Aldrich 98% purity, USA) was used in the mobile phase.

For separation and molecular detection, an ultra-high-performance liquid chromatograph was used with diode array detector (UHPLC-DAD, Agilent 1290 Infinity II, Waldbronn, Germany) equipped with a quaternary high-pressure pump, column oven and Halmilton (Lancaster, PA, USA) PRP-X100 anion exchange column (5 µm, 150 mm × 4.6 mm). Elementary detection and determination of the concentration of Cr species was performed in an inductively coupled plasma mass spectrometer (ICP-MS, Agilent 7900, Hachioji, Japan), using high purity argon. The ICP-MS was equipped with a collision cell to minimize spectral interference. An acid distiller (DST-1000, Savillex, Eden Prairie, MN, USA) was used for nitric acid (HNO_3_ 65% m v^−1^, VTEC, Indaiatuba, Brazil) purification.

An acrylic material was used to make a low-cost flow split (“T” piece), aiming the Cr speciation through the hyphenation of the HPLC-DAD and ICP-MS techniques. A three openings (“T” confluence) flow split was developed: an opening for the inlet of eluent/sample from the column and two openings for directing eluent/sample to the detectors (DAD and ICP-MS). The flow rate of the solutions from the outlet was adequately developed according to the quantitative sample need for each detector. Therefore, the DAD eluent inlet was 2/3 (two thirds) of the total flow coming from the column. To test the flow rate from each outlet, the water flow rate in the HPLC was set on 0.4 mL min^−1^. Then, the amount of eluent that came out of each tip of the “T” piece was collected in a 2 mL plastic tube for 5 min and weighed. Additionally, the length of the PEEK tubes (poly(ether-ether-ketone) was adjusted for simultaneous response in both detectors, DAD and ICP-MS.

### 2.2. Chemical Speciation of Chromium

The Cr(III) to be detected in the ultraviolet-visible (UV-Vis) spectrum must be in the form of [Cr(III)-EDTA]^−^. Thus, we prepared [Cr(III)-EDTA]^−^ complex from CrCl_3_ 6H_2_O salt in an aqueous solution with free EDTA in a 1:1 ratio. The CrCl_3_(6H_2_O) salt was weighed to obtain the mass equivalent of 100 mg L^−1^ of Cr(III) together with EDTA salt (100 mg L^−1^ of Cr(III)). The solution was then kept at 60–70 °C for 1 h and the pH was adjusted to 4 with NH_4_OH. The procedure used was adapted from Byrdy et al. [13] and Hamilton Company [14].

The calibration curves of Cr species were used for initial evaluation and application of the method in HPLC-DAD/ICP-MS hyphenation. The calibration standards were prepared in aqueous medium from sequential dilution of the standard solution Cr(VI) and the [Cr(III)-EDTA]^−^ complex at concentrations 0.1; 0.2; 0.3; 0.4; 0.5 mg L^−1^. NH_4_NO_3_ was used to prepare the mobile phase; the aqueous solution was adjusted to pH 7 with NH_4_OH (28% NH_3_ in water, ≥99.99% purity, Sigma Aldrich, USA), Table 1. The monitored wavelengths for [Cr(III)-EDTA]^−^ and Cr(VI) were 550 nm [15,16,17,18,19] and 371 nm [15,20], respectively. The calibration curve for the total determination of Cr was prepared in aqueous medium from the sequential dilution of the standard solution of Cr(VI) at concentrations 1; 5; 10; 50; 100 e 250 µg L^−1^ (Table 2).

### 2.3. Extraction of Chromium Chemical Species

The analysis was performed using 2 certified reference materials (CRMs) and one supplement. The Sugar cane leaf powder (CRM-Agro FC_012017) and natural water (1640a—National Institute of Standards and Technology (NIST)) were used to test the extraction and the recoveries/accuracy of the method (in triplicate). The supplement of Cr picolinate was analyzed in order to test the method in a pharmaceutical sample. The sample extraction of Cr species was based on Unceta et al. [5], Vacchina, Calle and Séby [22] and Zhang et al. [23]. For CRM natural water, no additional preparation was performed. For CRM sugar cane leaves and Cr picolinate supplement (containing Cr picolinate, caffeine and arginine), preparation was as follows: (1) about 200 mg of sample was weighed into a Falcon tube; (2) 10 g of 5 mM EDTA solution at pH = 7.5 (adjusted with NH_4_OH) was added; (3) sonication of the solution in ultrasonic bath for 20 min; (4) heating in a water-bath (45–60 °C) for 2 h and then cooling to room temperature; (5) centrifugation for 10 min at 4400 RPM (revolutions per minute); (6) filtration through a Sartorius Minisart^®^ hydrophilic syringe filter (0.2 µm, cellulose). For the supplement, the sample was diluted to 40 mL before centrifugation (step 5). The blanks were performed with the same procedure described except for the addition of sample.

The sample preparation to determine the total concentration of Cr in the extracts was adapted from Paniz et al. [24]. In triplicate, 1 mL of extract was added in a 15 mL plastic tube. The extract was dried in an oven at 80 °C for 2 h. Then, 5% w w^−1^ distilled nitric acid (HNO_3_) (65% w w^−1^, Synth, São Paulo, Brazil) was added. After 24 h pre-digestion, the samples were heated in a digester block at 90 °C for 4 h. After that, the volume was made up to 10 mL with ultra-pure water. The blanks were performed with the same procedure described except for the addition of sample. The operation conditions of the ICP-MS are described in Table 2. 

### 2.4. Figures of Merit and Chromatographic Parameters of the Method

The present work evaluated figures of merit and chromatographic parameters of the developed method. The calibration curves were evaluated for the linearity parameter, the coefficient of determination (R^2^). The limit of detection (LOD) and limit of quantification (LOQ) of the analyses were also evaluated for ICP-MS. These parameters were calculated through the average of the analysis of the blank values of the samples with the standard deviation in 10 replicates (*n* = 10) [25]; the blank of the extracts and the blank of total Cr determination were also analyzed. For LOD, the calculation was the mean concentration of the 10 replicates of blank plus 3 times the standard deviation; for LOQ, we considered 3.3 times the LOD.

The method performance was evaluated by applying two different columns of PRP-X100, on different days, from different solutions of mobile phase and calibration curve. One column was already used for other studies (in this column we set the flow rate of 0.5 mL) and a second column, brand new, operated with a flow rate of 0.45 mL. The time variation in which the dead volume appeared in the chromatogram during the chromatographic development was monitored, as this would influence the elution time of the other peaks.

Furthermore, the quality of the chromatographic conditions selected for the determination of Cr(III) and Cr(VI) was verified by chromatographic parameters, selectivity (ɑ) and asymmetry factor (As). The selectivity calculation can be performed from Equation (1), where k_2_ represents the retention factor of the second peak and k_1_ the factor of the first peak [26]. The asymmetry factor can be obtained through Equation (2), where B and A are the peak width measurements obtained from the retention time at 10% of the peak height, where B is the right band and A is the left band [27].

A = k_2_/k_1_ (1)

As = B/A(2)

## 3. Results and Discussion

### 3.1. Development of “T Piece” and Evaluation of the Cr Chemical Speciation

**Figure 1:**
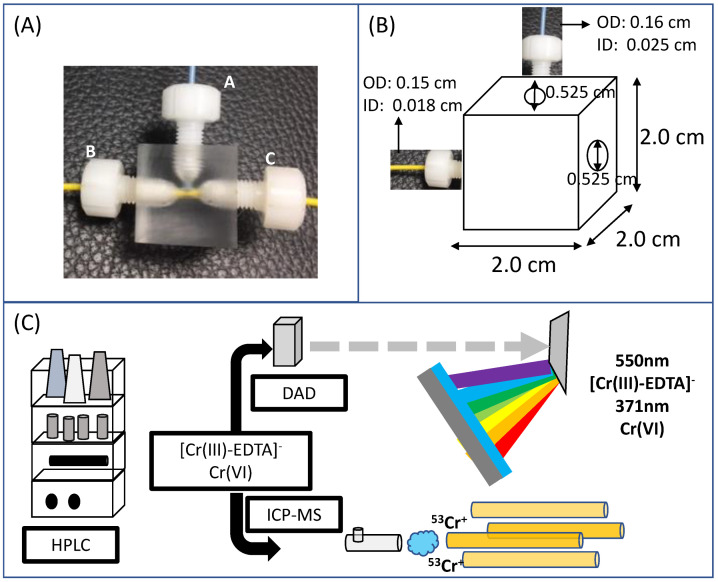
“T” piece developed for simultaneous hyphenation. (**A**): Acrylic cube developed (2 × 2 × 2 cm) for the hyphenation of the HPLC-DAD/ICP-MS. 1A/A: eluent/sample inlet from the HPLC column (capillary length 42 cm); (**A**,**B**): eluent/sample outlet (63%) to the DAD detector (capillary length 15 cm); 1A/C: eluent/sample outlet (37%) to the ICP-MS detector (capillary length 75.5 cm). (**B**): Detailed dimensions of the “T” piece. (**C**): Technical illustration of how each detector responds to the presence of Cr.

The HPLC-DAD/ICP-MS coupling was performed using a flow split (Figure 1). In this type of hyphenation, the eluent/sample is split simultaneously to both detectors. This is one of the advantages of this type of hyphenation in relation to the “tandem” type, in which the flow is unique and continuous between the detectors. The “T” piece developed in the present study was made of acrylic (inert and resistant material) and presented a targeting elution of 37% for ICP-MS (elemental detection) and 63% for DAD (molecular detection), simultaneously. In addition, the DAD is a non-destructive detector, which allows the recovery of the detected analytes, an impossible duty when using ICP-MS only or tandem DAD-ICP-MS, for instance.

**Figure 2:**
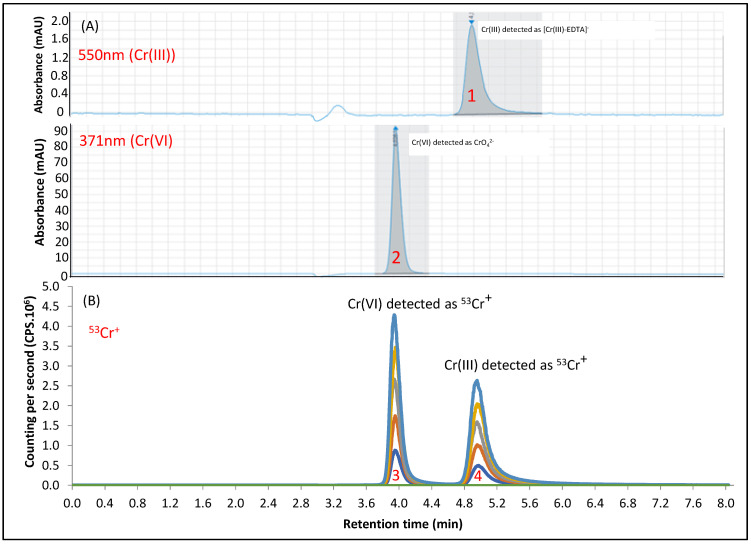
Chromatographic representation of Cr speciation analysis by HPLC-DAD/ICP-MS. (**A**): elution of [Cr(III)-EDTA]^−^ (peak 1) and Cr(VI) (peak 2) at 1 mg L^−1^, monitored wavelengths 550 nm and 371 nm, respectively. (**B**): ICP-MS chromatograms of calibration solutions for Cr(VI) (peak 3) and Cr(III) (peak 4) after injection in HPLC-DAD/ICP-MS. Note: Color/mg L^−1^: Orange/0 (blank); Dark blue/0.1; Red/0.2; Green: 0.3; Purple: 0.4; Light blue: 0.5.

For Cr speciation, Mihai et al. [28] evaluated different constitutions of the mobile phases (NH_4_NO_3_: 40 mM, 50 mM or 60 mM and; pH 6, 7, 8 or 9) to observe the association between the interconversion of Cr(VI)/Cr(III) and the pH. Interconversions occur because the stability of Cr is significantly dependent on pH; alkaline medium can oxidize Cr(III) to Cr(VI) and acidic ones can reduce Cr(VI) to Cr(III). The authors observed no interconversion, noticing only modifications in the peak’s profile and the separation. Finally, the authors concluded that the best separation was in pH = 8, using a mobile phase of NH_4_NO_3_ 50 mM. Sakai et al. [21] detected [Cr(III)-EDTA]^−^ and Cr(VI) in plastic toys using an anion exchange column and mobile phase composed of 75 mM HNO_3_ (pH = 7) in a flow rate of 0.8 mL min^−1^. Zhang et al. [23] used NH_4_NO_3_ 76 mM, pH = 7.2 in a flow rate of 1 mL min^−1^ aiming to evaluate the presence of Cr(VI) in a popular supplement of Cr (Cr-enriched yeast, CrY). The authors performed the separation using two units of anion exchange guard columns (Thermo Scientific Dionex IonPac AG7, 4 mm × 50 mm) at room temperature. According to Pechancová et al. [29], there were no previous studies available in the literature on the application of Cr speciation in biological samples. Only two previous studies had used HPLC-ICP-MS for chromium speciation in blood and urine. The method developed by the authors also used the Halmilton PRPX-100 anion exchange column (5 µm, 150 mm × 2.1 mm set at 21 °C) with an isocratic mobile phase solution containing NH_4_NO_3_ (30 mM), pH = 6. In the present study, a Halmilton PRP-X100 anion exchange column (150 mm × 4.6 mm, 5 µm particle size), widely used for arsenic and selenium chemical speciation, was applied (25 °C) for Cr separation. The mobile phase used was based on the studies of Zhang et al. [23] and Pechancová et al. [29] with modifications. We increased the concentration of NH_4_NO_3_ and decreased the pH for best separation and resolution, observing no interconversion of Cr-species. Figure 2 shows the chromatograms obtained by using the analysis conditions presented in Table 1 and Table 2. In Figure 2, we also observe that the retention times of Cr(III) and Cr(VI) are similar in both detectors.

**Figure 3:**
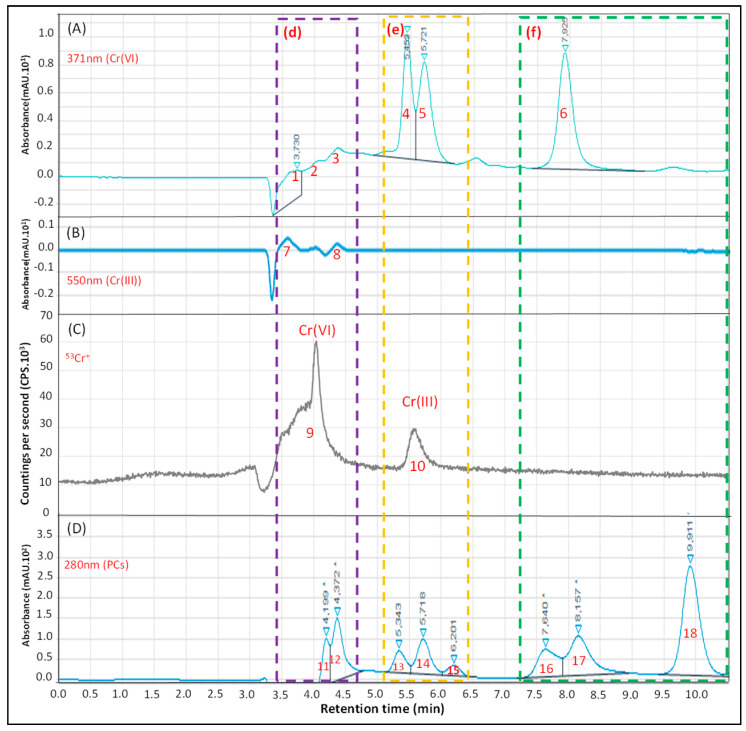
Analysis of the CRM Sugar cane leaf extract (CRM-Agro FC_012017) for Cr speciation by HPLC-DAD/ICP-MS. (**A**): 371 nm chromatogram for monitoring Cr(VI) (as CrO_4_^2−^). (**B**): 550 nm chromatogram for monitoring Cr(III) (as [Cr(III)-EDTA]^−^). (**C**): ^53^Cr isotope monitoring (as ^53^Cr^+^) in ICP-MS. (**D**): 280 nm chromatogram for monitoring possible biomolecules associated with Cr. 1 to 18: identified peaks. d, e and f: specific regions of the chromatograms.

**Figure 4:**
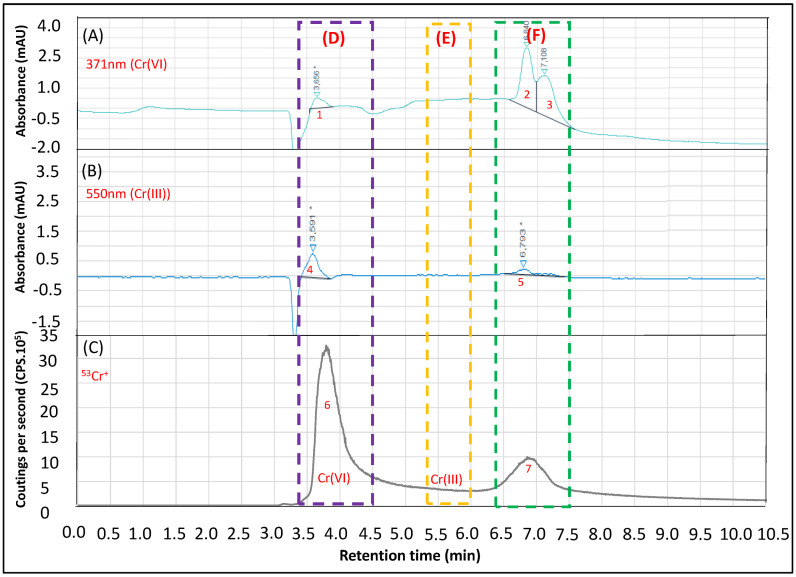
Analysis of the CRM natural water (1640a—NIST) for Cr speciation by HPLC-DAD/ICP-MS. (**A**): 371 nm chromatogram for monitoring Cr(VI) (as CrO_4_^2-^). (**B**): 550 nm chromatogram for monitoring Cr(III) (as [Cr(III)-EDTA]^−^). (**C**): ^53^Cr isotope monitoring (as ^53^Cr^+^) in ICP-MS. 1 to 7: identified peaks. D, E and F: specific regions of the chromatograms.

Before injecting a real sample into HPLC-DAD/ICP-MS, the extract of the reference material sugar cane leaves (CRM-Agro FC_012017) was digested and the total concentration of Cr was determined by ICP-MS. The recovery of total Cr in the extract of sugar cane leaves was 85.4% (0.317 ± 0.058 mg kg^−1^), compared to the certified reference value (0.371 ± 0.023 mg kg^−1^). The same extract, without acid digestion, was injected in HPLC-DAD/ICP-MS (Figure 3, discussed below). The natural water (1640a—NIST) was directly injected into ICP-MS without sample treatment and was analyzed by ICP-MS. The recovery of Cr was 99.7% (40.41 ± 2.88 μg L^−1^), compared to the reference value (40.54 ± 0.30 μg L^−1^). The same original solution was injected into HPLC-DAD/ICP-MS (Figure 4, discussed below).

### 3.2. Evaluation of the Hyphenation Injection of Real Samples in the HPLC-DAD/ICP-MS

The analyses described in this study were performed in the acquisition mode total ion chromatogram (TIC) for the ICP-MS. The equipment performed a continuous reading of the signal strength at the *m*/*z* 53 (^53^Cr^+^) selected for a certain period of time (the chromatographic run) [30], thus obtaining peaks of a chromatogram observed in the technique of chromatographic detection (DAD), making easier the interpretation and correlation of the results obtained. 

Figure 3 presents the results obtained for Cr speciation in the CRM sugar cane leaves by the hyphenated system HPLC-DAD/ICP-MS. It is possible to observe six peaks (1 to 6) at the 371 nm (Cr(IV) wavelength (Figure 3A). For the 550 nm (Cr(III)) wavelength (Figure 3B), it is possible to observe two small peaks (7 and 8). Regarding the detection by ICP-MS (Figure 3C), a poorly defined peak is observed from the elution time of 3.4 to 4.6 (peak 9, d region (purple)) that may be associated with the presence of Cr(VI) due to the increasing signal (peaks 1 to 3 in Figure 3A) and polyatomic interferences in ICP-MS caused by the presence of EDTA (high amounts of carbon in plasma forming ^4^°Ar^12^C^+^/^40^Ar^13^C^+^, the most common interferents for ^52^Cr+/^53^Cr^+^, respectively) once this peak is observed in blank at smaller intensity. Also, Figure 3D (DAD at 280 nm) suggests the presence of phytochelatins (PCs) (peaks 11 and 12, d region), usually associated with the accumulation of metals and present in plants [11,31]. In the e region (yellow, Figure 3A) it is possible to observe the detection of peaks 4 and 5 and in ICP-MS the peak 10 (Figure 3C). However, no peak was observed in Figure 3B. In the same region, at the 280 nm wavelength (Figure 3D), we observed three peaks (13, 14 and 15) indicating the presence of biomolecules (possibly PCs) indicating the presence of Cr. In ICP-MS, it was not possible to detect the presence of ^53^Cr^+^ after 7.3 min (f region, green). Therefore, the peaks 6, 16, 17 and 18 observed in Figure 3A,D may be associated with biomolecules present in the leaves’ extract without Cr in their composition. The importance of chemical speciation with double detection is demonstrated here. Further experiments are necessary to better characterize and understand the chemical composition of a given extract by using a new mass detector associated with a molecular structure [10,11]. 

It is known that increasing soil pH and cation exchange capacity increases the adsorption of Cr(III) in soils. Cr(III) is considered stable due to the absence of natural oxidants, such as MnO_2_, an effective agent for the interconversion Cr(VI) to Cr(III). Additionally, Cr(III) in neutral pH ranges (4–9), typical of natural waters, is present in insoluble form as relatively inert precipitates, with easy adsorption in solid phases. On the other hand, Cr(VI) presents high water and soil mobilities [2,32,33]. Figure 4 presents the results obtained for the CRM natural water NIST 1640a. It is possible to observe in the D region (purple) the peaks 1, 4 and 6. For Figure 4A,C, we observed two peaks (1 and 6, respectively) associated to Cr(VI). Peak 4 (Figure 4B) is possibly another trace element/concomitant with absorbance at 550nm present in the water sample. In the E region (yellow), the retention time of Cr(III), we did not observe any peak regarding the *m*/*z* 53 (Figure 4C) or absorbance at 550 nm. At F region (green), it is possible to identify a peak (7, Figure 4C) with absorbances at 371 nm (peaks 2 and 3, Figure 4A) and 550 nm (peak 5, Figure 4B). The interpretation of this region of the chromatograms leads us to believe that peak 7 is a double ionized isotope of another element (such as ^105.9035^Ag^++^: *m*/*z* = 52.9517, quadrupole resolution 0.5 u.m.a., from 52.5 to 53.5 u.m.a. for *m*/*z* 53) or polyatomic interference (at *m*/*z* 53) of the elements at the highest concentrations in the NIST 1640a such as Si, Ca, Na, K and Mg (^40^Ca^13^C^+^, ^40^Ca^12^C^1^H^+^, ^40^K^13^C^+^, ^40^K^12^C^1^H^+^, ^23^Na^30^Si^+^, ^24^Mg^29^Si^+^, ^24^Mg^28^Si^+^), separated by the anion exchange column. Once there are many potentially interferences, more studies (such as the testing of reaction/collisional gases; spiking the solution of the elements, etc.) are necessary to investigate the origin of the observed peaks.

**Figure 5:**
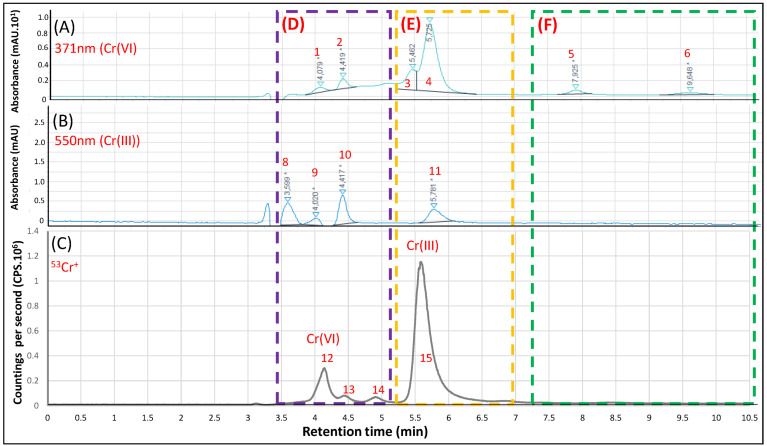
Analysis of the Cr-picolinate supplement for Cr speciation by HPLC-DAD/ICP-MS. (**A**): 371 nm chromatogram for monitoring Cr(VI) (as CrO_4_^2−^). (**B**): 550 nm chromatogram for monitoring Cr(III) (as [Cr(III)-EDTA]^−^). (**C**): ^53^Cr isotope monitoring (as ^53^Cr^+^) in ICP-MS. Numbers 1 to 15: identified peaks. D, E and F: specific regions of the chromatograms to be observed.

Cr(III) can be consumed, among other ways, through dietary supplements, with chromium picolinate being widely used due to its improved absorption and intracellular uptake [28]. Figure 5 presents the results obtained in the hyphenated HPLC-DAD/ICP-MS for the analysis of Cr speciation in a commercial food supplement of Cr picolinate containing 250 µg of Cr per capsule. In the D region (purple), it is possible to observe eight peaks (1–2; 8–10; 12–14). Considering the retention time, Cr(VI) is observed in Figure 5C (peak 12, ICP-MS) and Figure 5A (peak 1, DAD). Also, we observed the presence of other peaks of compounds containing Cr (13 and 14): peak 13 with absorbance at 371 nm (peak 2, Figure 5A) and 550 nm (peak 10, Figure 5B) and peak 14 with no correspondence absorbance. In region E (yellow), the presence of Cr(III) is evident (Figure 5C, peak 15), with absorbance at 550 nm (peak 11) and also 371 nm (peaks 3 and 4). The F region (green) presents only two peaks (5 and 6) with absorbance at 371 nm and no presence of detectable concentrations of Cr (no peaks in Figure 5C). Due to the non-specificity of the absorbance, it is necessary to apply more accurate and specific detectors such as high-resolution mass spectrometers (such as Orbitrap^R^ [10]) to improve the identification of these potential Cr-containing biomolecules and control the quality of supplements destined for human consumption. 

Amayo et al. [10] also studied simultaneous hyphenation. They hyphenated the HPLC to an ICP-MS and ESI-MS, both high resolution techniques. During the study, arsenolipid compounds were evaluated in samples of the meal of capelin fish (*Mallotus villosus*) without the use of species-specific standards. They concluded that the identification of arsenic-containing compounds occurs directly, even without the presence of compounds of similar chromatographic behavior, using the molecular weight accuracy of the high-resolution mass spectrometers. The authors also state that this type of hyphenation can be applied to any element detected by ICP-MS, as demonstrated in this present study. Methods involving voltammetry and Cr-speciation are of paramount importance for the analysis of water [34,35,36]. Compared to voltammetry, HPLC-DAD/ICP-MS presents important advantages such as multielement analysis and the possibility of introducing higher complex matrices containing innumerous biomolecules containing Cr and/or other elements [10,11]. As far as we know, this is the first study about the analytical methods for Cr speciation that allow the simultaneous detection of molecules and elements in a single sample injection. Finally, this method has enormous potential to be applied for other elements present in biomolecules such as Fe, Co, Mg, Se.

### 3.3. Evaluation of Figures of Merit and Chromatographic Parameters

**Figure 6:**
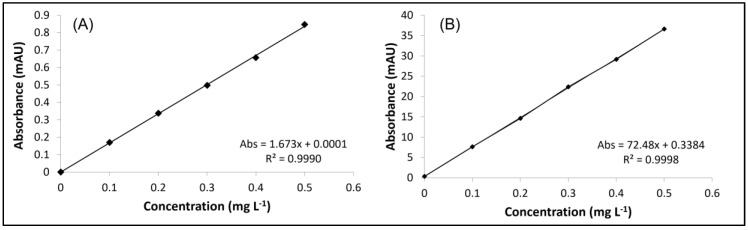
Calibration curve in HPLC-DAD for analysis of Cr speciation by HPLC-DAD/ICP-MS. (**A**): [Cr(III)-EDTA]^−^, wavelength 550 nm. (**B**): Cr(VI), monitored wavelength 371 nm.

**Figure 7:**
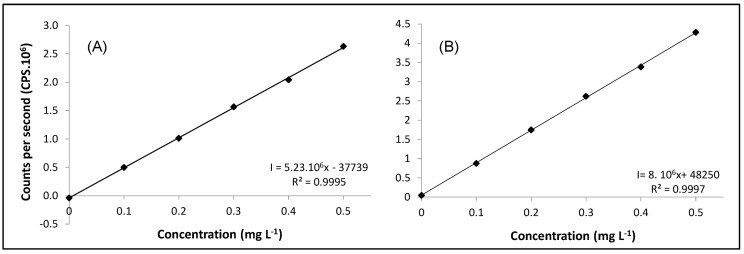
Calibration curve performed in ICP-MS for Cr speciation by HPLC-DAD/ICP-MS. (**A**): [Cr(III)-EDTA]^−^ detected at *m*/*z* 53. (**B**): Cr(VI) detected at *m*/*z* 53.

**Figure 8:**
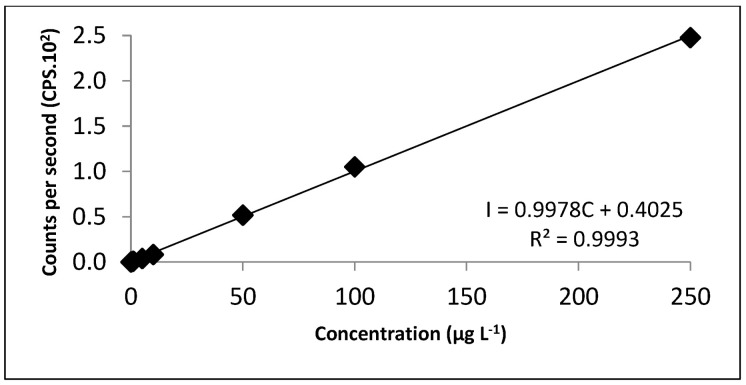
Calibration curve performed in ICP-MS to determine the total concentration of Cr in the samples. The equipment operating conditions are described in Table 2. Calibration curve equation: I is the intensity (CPS) and C is the concentration (µg L^−1^).

The LOD can be defined as the detected concentration that can be differentiated from the noise of the analytical signal and the LOQ can be defined as the lowest quantified concentration [37]. The values obtained for both blanks were below the values of the calibration curve used to determine the concentration of Cr in the extracts of the samples. The LOD and LOQ of the blank of the extracting solution were 0.239 µg L^−1^ and 0.415 µg L^−1^, respectively. The blank of the total determination, the values obtained for LOD and LOQ, were 0.157 µg L^−1^ and 0.261 µg L^−1^, respectively. Figure 6 shows the calibration curves obtained on HPLC-DAD for Cr speciation in HPLC-DAD/ICP-MS hyphenation. Aqueous solutions containing [Cr(III)-EDTA]^−^ and Cr(VI) were diluted at concentrations of 0.10, 0.20, 0.30, 0.40 and 0.50 mg L^−1^ of each of the species. Both presented R^2^ ≥ 0.9990. Figure 7 presents the calibration curve for ICP-MS for Cr speciation after the injection in the HPLC-DAD/ICP-MS. The response obtained by the MS detector refers to the total concentration of Cr, determined by the monitoring of the ^53^Cr isotope. Both curves for the species of [Cr(III)-EDTA]^−^ and Cr(VI) showed R^2^ ≥ 0.9990. Figure 8 shows the calibration curve obtained in ICP-MS for the determination of total Cr in samples of sugar cane and water. The standard aqueous solution containing Cr(VI) at 100 mg L^−1^ was diluted to a concentration of 1, 5, 10, 50, 100 and 250 µg L^−1^. The curve presented R^2^ ≥ 0.9990.

According to Ribeiro et al. [37], the method’s robustness can be defined as the variation of several analytical parameters and the method’s ability to resist these small changes. The results between different Hamilton PRP-X100 anion exchange columns with different usage times did not show significant variations (<8%) during the calibration curve, approximately 12 s, an expected variation due to the column lifetime. Since the new column has more interaction sites with the mobile phase, the response time occurred 12 s later than that observed for the column already used for other studies of the research group.

Selectivity can be defined as the method’s ability to separate the analyte of interest from other components that may be present in the sample [26]. The calculation was performed from the ’k’ values presented for the analytes of interest and the value obtained was 1.94. From this value, it is possible to affirm that there is a satisfactory separation between the peaks, since the minimum recommended for this parameter is values above 1 [26].

The asymmetry factor has a recommended acceptance criterion of 0.9 to 1.2 and is directly related to chromatographic efficiency; this interval range indicates low or no tail influence on the chromatographic peaks [26,27]. The values obtained for the peaks from the calibration curves for [Cr(III)-EDTA]^−^ and Cr(VI) were 1.2 and 1.4, respectively. The values can be considered satisfactory as they are below 1.5 and indicate low tail influence on the peaks [38]. 

## 4. Conclusions

The low-cost flow split was shown to be promising for the developed method, allowing the responses of both detectors (DAD and ICP-MS), simultaneously, without interfering in the elution, performing excellent resolution of the chromatographic profiles. Additionally, the use of the flow splitter allowed the collection of sample fraction directed after the DAD for future complementary analyses by other analytical techniques, since this is a non-destructive detector.

The coupling of the techniques through the use of a flow splitter allowed an increase in the amount of information obtained in a more practical way (with just one injection of the sample) in the same time interval. The CRMs and the Cr supplement presented simultaneous molecular and elemental results, enabling the combination of information with one sample injection.

This initial study of Cr speciation in simultaneous monitoring is an important step for future analysis to obtain information about Cr-containing biomolecules, saving time and resources. There are still many gaps to be filled, however, regarding the development of bioanalytical methods. 

## Figures and Tables

**Table 1:** Experimental conditions evaluated for HPLC. Isocratic elution, according to Sakai et al. [21].

Column	Hamilton PRP-X100 (5 µm, 150 mm × 4.6 mm)
Mobile phase	NH_4_NO_3_ 225 mM at pH = 7
Mobile phase flow	0.45–0.5 mL min^−1^
Elution type	Isocratic
Race time	8 min
Rinsing	3 wash cycles
Injection volume	30 µL
Quantification	Peak area

**Table 2:** Experimental conditions for ICP-MS.

Radio frequency power	1550 W
Gas flow (Ar)	15 L min^−1^
Nebulizing gas flow	0.9 L min^−1^
Interface	Nickel cones
Sampling cone	0.9 mm
Extractor cone (skimmer)	0.45 mm
Nebulizer	Micro Mist^TM^
Monitored Isotope	^53^Cr

## Data Availability

Data available on request due to restrictions e.g., privacy.
